# Graph Convolutional Networks for multi-modal robotic martial arts leg pose recognition

**DOI:** 10.3389/fnbot.2024.1520983

**Published:** 2025-01-20

**Authors:** Shun Yao, Yihan Ping, Xiaoyu Yue, He Chen

**Affiliations:** ^1^Department of Public Instruction, ChangJiang Polytechnic of Art and Engineering, Jingzhou, China; ^2^School of Computer Science, Northwestern University, Evanston, IL, United States; ^3^School of Physical Education, Hubei University of Science and Technology, Xianning, China; ^4^College of Physical Education, Sangmyung University, Seoul, Republic of Korea

**Keywords:** martial arts pose recognition, spatial-temporal graph encoding, Graph Convolutional Networks, action-specific attention, self-supervised learning

## Abstract

**Introduction:**

Accurate recognition of martial arts leg poses is essential for applications in sports analytics, rehabilitation, and human-computer interaction. Traditional pose recognition models, relying on sequential or convolutional approaches, often struggle to capture the complex spatial-temporal dependencies inherent in martial arts movements. These methods lack the ability to effectively model the nuanced dynamics of joint interactions and temporal progression, leading to limited generalization in recognizing complex actions.

**Methods:**

To address these challenges, we propose PoseGCN, a Graph Convolutional Network (GCN)-based model that integrates spatial, temporal, and contextual features through a novel framework. PoseGCN leverages spatial-temporal graph encoding to capture joint motion dynamics, an action-specific attention mechanism to assign importance to relevant joints depending on the action context, and a self-supervised pretext task to enhance temporal robustness and continuity. Experimental results on four benchmark datasets—Kinetics-700, Human3.6M, NTU RGB+D, and UTD-MHAD—demonstrate that PoseGCN outperforms existing models, achieving state-of-the-art accuracy and F1 scores.

**Results and discussion:**

These findings highlight the model's capacity to generalize across diverse datasets and capture fine-grained pose details, showcasing its potential in advancing complex pose recognition tasks. The proposed framework offers a robust solution for precise action recognition and paves the way for future developments in multi-modal pose analysis.

## 1 Introduction

Leg pose recognition in martial arts performance has become an essential task for applications in sports analysis, rehabilitation, and interactive training systems, enabling both quantitative assessment and real-time feedback for practitioners (Rodomagoulakis et al., 2016). Recognizing leg movements in martial arts requires not only capturing the fine-grained dynamics of specific actions but also understanding complex sequences where stability, coordination, and speed are crucial. With the growing popularity of AI-driven sports technology, the demand for robust and accurate pose recognition models has increased significantly, as these models not only enhance the understanding of martial arts techniques but also contribute to improving training safety and effectiveness (Mollaret et al., 2016). Furthermore, advanced recognition techniques allow for automatic performance evaluation and help practitioners achieve precision, consistency, and progression in their movements. This task's complexity and relevance make it a critical area for innovation in action recognition and pose estimation (Van Amsterdam et al., 2022).

To address the challenges of leg pose recognition, early approaches relied on symbolic AI and knowledge representation techniques. These methods used expert-crafted rules and domain-specific knowledge to model martial arts poses. By defining key features such as joint angles, positions, and limb alignments, these rule-based systems could classify static poses and limited sequences with acceptable accuracy (El-Ghaish et al., 2017). Symbolic methods often represented poses through knowledge graphs or ontology-based systems that encoded anatomical relationships and biomechanical principles. While effective for static poses or simple actions, these methods were limited in their ability to generalize to diverse or dynamic martial arts movements (El-Ghaish et al., 2017). They struggled with real-time application due to the time-intensive process of rule crafting, and their lack of adaptability meant they could not accommodate the variations inherent in different practitioners' performances. To improve the flexibility and scalability of these models, researchers turned toward data-driven techniques (Romaissa et al., 2021).

To overcome the limitations of rule-based methods, data-driven approaches grounded in machine learning were introduced. These models relied on statistical learning techniques to infer patterns directly from labeled pose data, marking a transition from rigid, rule-based frameworks to adaptable models that could learn from examples. Support Vector Machines (SVM; Verma et al., 2020), k-Nearest Neighbors (k-NN; Duhme et al., 2021), and Hidden Markov Models (HMM; Cruz et al., 2016) became popular for action classification tasks, and Principal Component Analysis (PCA; Gaonkar et al., 2021) was used to reduce the dimensionality of joint data, improving efficiency. These models successfully captured patterns in labeled datasets and offered better generalization than symbolic AI. However, their effectiveness depended heavily on the quantity and quality of labeled data, making it difficult to scale for diverse martial arts actions that exhibit intricate temporal dependencies. While they were more adaptable than symbolic methods, data-driven models often lacked the ability to capture deep contextual relationships, limiting their accuracy for highly dynamic or complex martial arts leg poses. To enhance robustness in handling varied action sequences, research began shifting toward deep learning models capable of more intricate feature extraction.

Addressing the limitations of data-driven methods, the field advanced to deep learning and pretrained models, which offered unprecedented improvements in feature representation and action recognition. Convolutional Neural Networks (CNNs; Zhang et al., 2019) and Long Short-Term Memory (LSTM; Bednarek et al., 2020) networks became the standard for learning spatial and temporal features from video sequences and skeleton data, respectively. Graph Convolutional Networks (GCNs; Naik and Kumar, 2011) further enabled researchers to model joint interactions through graph structures, achieving state-of-the-art accuracy by capturing both the spatial configuration of joints and the temporal progression of actions. Pretrained models, such as transformer-based architectures, offered new possibilities by leveraging large datasets to learn generalized representations that could be fine-tuned for martial arts pose recognition (Naik and Kumar, 2009). Despite their high accuracy, these models were computationally intensive and required substantial labeled data for effective fine-tuning. Additionally, pretrained models lacked explicit mechanisms for integrating domain-specific knowledge, often resulting in reduced interpretability. To address these issues, our method introduces a framework that leverages graph structures while incorporating attention mechanisms and self-supervised tasks to enhance temporal consistency and efficiency.

To overcome the limitations of previous methods, we propose PoseGCN, a specialized framework for martial arts leg pose recognition that integrates spatial, temporal, and contextual features to capture the complexity of martial arts movements. Unlike traditional models, PoseGCN's architecture is designed to handle rapid transitions and detailed joint positioning, key to accurately identifying martial arts actions. Central to PoseGCN is its spatial-temporal graph encoding module, which represents each pose sequence as a graph where nodes denote joints and edges capture spatial and temporal dependencies, allowing the model to recognize subtle, context-dependent leg movements. Additionally, PoseGCN introduces an action-specific attention mechanism that dynamically assigns importance to key joints based on the action context. For instance, in a high kick, the model prioritizes the hip and knee joints, while in balanced stances, it shifts focus to the ankle and foot, enhancing the model's accuracy in differentiating between similar poses. Finally, PoseGCN incorporates a self-supervised pretext task that improves its ability to capture temporal dependencies by learning frame order without extensive labeled data. Together, these components make PoseGCN not only accurate and generalizable but also robust across diverse datasets, setting a new benchmark in leg pose recognition for martial arts applications.

PoseGCN offers several advantages over traditional and modern approaches:

- It introduces an action-specific attention mechanism that dynamically allocates importance to joints based on the action context, enhancing accuracy in recognizing complex leg poses.
- The model's spatial-temporal graph encoding efficiently captures joint dynamics, making it applicable across various martial arts poses and adaptable to real-time scenarios.
- Experimental results demonstrate that PoseGCN achieves superior accuracy and F1 scores on benchmark datasets, establishing it as a robust solution for leg pose recognition tasks.

## 2 Related work

### 2.1 Human pose estimation and recognition techniques

Human pose estimation and recognition have been extensively studied in computer vision, with approaches evolving from traditional image processing techniques to advanced deep learning models. Early methods focused on handcrafted features such as Histogram of Oriented Gradients (HOG; Wang et al., 2024) and Scale-Invariant Feature Transform (SIFT), which demonstrated some success in static pose estimation but lacked robustness for complex or dynamic human actions. With the advent of deep learning, Convolutional Neural Networks (CNNs) and Recurrent Neural Networks (RNNs; Zhu et al., 2024) became widely adopted, offering improved performance on human pose estimation tasks. CNN-based models, including Stacked Hourglass Networks and OpenPose, have proven effective in detecting body landmarks in 2D images, laying the groundwork for subsequent action recognition tasks. However, these approaches often fall short when applied to dynamic actions, as they lack temporal modeling capabilities necessary to capture the intricacies of motion over time. To address this, methods such as Long Short-Term Memory (LSTM) networks and Temporal Convolutional Networks (TCNs) have been introduced, allowing for temporal sequence learning. Although these architectures enhance temporal awareness, they still struggle with spatial dependencies between joints, especially in complex multi-joint movements (Dhiman and Vishwakarma, 2020). Recent advancements have focused on Graph Convolutional Networks (GCNs), which use graph structures to model joint dependencies, offering a more robust framework for capturing both spatial and temporal patterns in human actions. Despite their effectiveness, many GCN models are limited in their ability to generalize across varying contexts, as they often lack attention mechanisms or self-supervised learning modules to enhance robustness, highlighting areas for further research in complex pose recognition tasks (Jin et al., 2024).

### 2.2 Multimodal action recognition

Multimodal action recognition integrates information from multiple data sources, such as RGB video, depth sensors, and inertial measurements, to improve accuracy in complex action classification tasks (Naik and Kumar, 2011). Traditional approaches often rely on early or late fusion strategies, combining features from different modalities either at the input level or decision level. Early methods integrated RGB and depth data to address occlusion and depth ambiguities, enhancing robustness in challenging settings. However, with the increasing availability of wearable devices and multimodal datasets, recent research has incorporated additional data from accelerometers, gyroscopes, and electromyography (EMG) sensors, enriching the action representation (Naik and Kumar, 2009). Deep learning has further enabled end-to-end fusion models, where convolutional and recurrent layers process multimodal data concurrently. Techniques such as Multimodal Transformer Networks and Cross-Modal Attention have emerged, allowing networks to dynamically adjust the importance of each modality based on context. While these methods yield strong performance in controlled environments, they often struggle with noisy or incomplete data, a common challenge in real-world applications. To address this, methods incorporating self-attention mechanisms and graph-based models for multimodal data have been proposed, enabling adaptive feature fusion across modalities (Dhiman et al., 2021). PoseGCN builds on this foundation by integrating spatial-temporal graphs and modality-specific attention layers, designed to dynamically emphasize key joints and adapt to varying modality importance, offering improved performance in complex actions such as martial arts leg pose recognition. Sharma et al. (2022) proposed a convolutional neural network method based on partial spatial-temporal attention, which improved the accuracy of action recognition by paying attention to different parts of the human body. In contrast, PoseGCN adopts a spatial-temporal graph representation structure based on a graph convolutional network (GCN), which can more accurately capture the dynamic relationship between joints. In addition, the attention mechanism of PoseGCN can dynamically adjust the weights of key joints according to different action scenarios, making it more adaptable under complex posture changes.

### 2.3 Self-supervised learning in pose estimation and action recognition

Self-supervised learning (SSL) has gained traction in action recognition and pose estimation due to its ability to learn robust representations from unlabeled data. In SSL, models are trained on pretext tasks, where labels are generated automatically to capture inherent structures in the data. Popular pretext tasks in action recognition include predicting the order of frames, learning motion dynamics, and reconstructing spatial arrangements (Dhiman and Vishwakarma, 2017), which help models learn temporal and spatial dependencies without relying on labeled data. SSL has been particularly beneficial for pose estimation, as it allows models to capture joint correlations and motion patterns even in the absence of annotated poses (Naik et al., 2015). Techniques like temporal shuffling, frame prediction, and geometric transformation prediction are widely used for SSL in pose-related tasks (Jin et al., 2023). Recent advancements include the use of contrastive learning, where models maximize agreement between augmented versions of the same action sequence while minimizing similarity with other sequences. This approach enables models to learn distinct features for each action type, enhancing generalization across datasets (Huang et al., 2021). However, SSL in pose estimation remains challenging due to the difficulty in defining effective pretext tasks that capture both spatial and temporal dependencies. PoseGCN incorporates a self-supervised pretext task involving frame order prediction, which enhances temporal consistency in its learned representations, making it more robust for downstream tasks like action classification. This use of SSL not only reduces dependency on labeled data but also contributes to improved temporal awareness and generalization in complex action sequences (Dhiman et al., 2019). Sharma et al. (2023) utilizes Shapley values to guide action recognition under long-tail distribution, focusing on improving accuracy in the presence of uneven data distribution. PoseGCN, by incorporating self-supervised learning modules and sparse coding strategies, enhances model performance on long-tail data, reducing dependence on balanced data distribution and demonstrating robustness in recognizing rare actions. These comparisons further underscore PoseGCN's computational efficiency and generalization capabilities in complex action recognition scenarios.

### 2.4 Multi-view learning and abnormal action recognition

In the field of pose recognition, achieving robust performance in multi-view invariance, sparse coding, self-supervised learning, and abnormal action recognition is essential. To enhance action recognition across different views, a study proposed a skeleton action learning approach based on motion retargeting, which extracts generalized skeleton features from various perspectives to address view changes. This approach inspires our incorporation of multi-view data augmentation and view invariance learning in the PoseGCN model (Yang et al., 2024). Sparse coding also plays a significant role in feature extraction. A study introduced a sparse-coded composite descriptor for recognizing human activity in high-dimensional feature spaces (Singh et al., 2022). By retaining only critical features, this method achieves efficient action recognition, serving as a reference for our use of sparse attention mechanisms in PoseGCN to enhance computational efficiency and model generalization (Dhiman and Vishwakarma, 2024). Recently, self-supervised learning has gained attention for handling incomplete sequences. A study applied self-supervised techniques to learn action representations from incomplete spatio-temporal skeleton sequences, demonstrating the potential to obtain meaningful representations from fragmented spatial-temporal features under data incompleteness or missing labels. This research provides theoretical support for our self-supervised learning module in PoseGCN, reducing dependency on labeled data (Zhang et al., 2024). For abnormal action recognition, one study proposed a robust framework based on R-transform and Zernike moments, effectively addressing abnormal action recognition in depth videos, particularly in terms of stability and accuracy in local feature processing. This work inspired us to introduce a key joint attention mechanism in PoseGCN to better distinguish normal from abnormal actions. Additionally, another study used histogram-oriented gradients and Zernike moments to achieve high-dimensional abnormal action recognition, identifying complex patterns in high-dimensional spaces, which offers technical guidance for efficient feature encoding in PoseGCN.

## 3 Methodology

### 3.1 Overview of our network

In this work, we propose an innovative framework for multimodal robotic martial arts leg pose recognition, leveraging Graph Convolutional Networks (GCNs) to enhance the integration of diverse data modalities, including spatial, temporal, and action-specific features. Our proposed model comprises several interconnected modules that process multimodal inputs to capture complex leg pose dynamics with high accuracy, particularly in challenging, fast-paced martial arts scenarios. The primary data flow begins with pose extraction from multi-sensor data inputs, which are then organized into graph structures. These structures feed into a specially designed Part-Level GCN, which emphasizes joint and segment-level relations, enhancing the representation of subtle pose variations. Furthermore, this architecture integrates contextual action information, enabling improved accuracy in distinguishing similar yet distinct leg positions and movements.

First, the computational complexity of PoseGCN mainly comes from graph convolution operations and self-attention mechanisms. The computational complexity of graph convolution is usually proportional to the number of nodes in the graph and the connection density of adjacent nodes. To reduce this complexity, we adopt a hierarchical structure in PoseGCN to divide the joints into local subgraphs, thereby reducing the overhead of full-graph computation. This can effectively capture local motion features and reduce the overall computational burden. Second, the self-attention mechanism is used in PoseGCN to dynamically adjust the weights of key joints, and the computational complexity increases linearly with the number of nodes. To reduce the computational burden, we adopt a sparse attention matrix and parameter sharing strategy in our implementation to reduce unnecessary computations and prune the attention weights, thereby reducing the requirements for memory and computational resources while maintaining model accuracy. Finally, considering the dependence of deep learning models on a large amount of labeled data, PoseGCN introduces a self-supervised learning module to pre-train the model with unlabeled data so that robust feature representation can be obtained even when there is insufficient labeled data. This self-supervised method not only reduces the dependence on labeled data, but also speeds up the convergence of the model to a certain extent, further reducing computational overhead.

Our method is structured as follows: Section 3.2 introduces the foundational preliminaries, where we mathematically formulate the leg pose recognition problem within a multimodal GCN context. In Section 3.3, we describe the proposed dynamic feature extraction and integration module, detailing its role in handling temporal variations and multimodal data alignment. Section 3.4 presents the novel learning strategy we implement, which utilizes prior knowledge specific to martial arts actions. This strategy facilitates efficient feature learning and optimizes the GCN for rapid adaptation to new leg poses and movements. Collectively, these sections illustrate a cohesive framework designed to achieve robust, high-accuracy recognition of martial arts leg poses through multimodal GCN enhancements.

### 3.2 Preliminaries

To effectively address the problem of leg pose recognition in a multimodal martial arts context, we begin by formulating the recognition task as a multimodal graph learning problem. Let us define a set of skeleton data $D={\left\{\left(X_{i},y_{i}\right)\right\}}_{i=1}^{N}$ where *X*_*i*_ represents the *i*-th sequence of joint data from the multimodal sensors, and *y*_*i*_ denotes the corresponding class label for the martial arts pose. Each sequence *X*_*i*_ consists of multiple frames, each capturing the 3D coordinates of a set of key joints across time.

Each frame in the sequence is represented as a graph *G* = (*V, E*), where *V* is the set of nodes corresponding to key joints, and *E* represents edges that encode anatomical connections and/or action-specific dependencies between joints. The pose recognition task aims to classify a given sequence *X* into one of the predefined martial arts leg pose categories based on the multimodal joint data and learned graph representations.

For each frame *t* in sequence *X*_*i*_, we define a graph *G*_*t*_ = (*V*_*t*_, *E*_*t*_), where each node *v*∈*V*_*t*_ is associated with a feature vector $f_{v}^{t}$ capturing joint coordinates and motion characteristics derived from multimodal sensors. Let $p_{v}^{t}=\left[x_{v}^{t},y_{v}^{t},z_{v}^{t}\right]$ denote the spatial coordinates of joint *v* at time *t*. Additionally, velocity and acceleration vectors ${\text{v}}_{v}^{t}$ and ${\text{a}}_{v}^{t}$ are computed to incorporate temporal dynamics. The edge set *E*_*t*_ is constructed by defining connections between anatomically or functionally related joints, forming a graph structure that captures both spatial and dynamic correlations in martial arts poses.

Given a sequence of graphs ${\left\{G_{t}\right\}}_{t=1}^{T}$, the objective is to learn a function F:G→Y, where G is the space of graph-structured input sequences, and Y is the set of pose labels. Each graph *G*_*t*_ is processed using graph convolutional layers, which aggregate information from neighboring nodes to capture spatial dependencies. The convolution operation on node *v* in frame *t* can be defined as:


(1) $$\begin{array}{l} h_{v}^{\left(l+1\right)}=\sigma \left(\underset{u\in N\left(v\right)}{\sum }W^{\left(l\right)}h_{u}^{\left(l\right)}+b^{\left(l\right)}\right), \end{array}$$

where $h_{v}^{\left(l\right)}$ represents the hidden state of node *v* at layer *l*, *W*^(*l*)^ is a trainable weight matrix, *b*^(*l*)^ is a bias term, and σ is a non-linear activation function. This formulation allows the model to capture localized patterns of movement and spatial correlations among joints, which are essential for distinguishing leg poses in martial arts.

To capture temporal dependencies across frames, we incorporate temporal convolution or recurrent mechanisms over the graph-structured data. Let $H^{\left(t\right)}={\left\{h_{v}^{\left(L\right)}\right\}}_{v\in V_{t}}$ denote the output node features at the final layer *L* for frame *t*. A temporal model T is then applied across ${\left\{H^{\left(t\right)}\right\}}_{t=1}^{T}$ to model frame-to-frame dependencies:


(2) $$\begin{array}{l} H_{\text{temporal}}=T\left(H^{\left(1\right)},H^{\left(2\right)},\ldots ,H^{\left(T\right)}\right), \end{array}$$

where T can be implemented as a temporal convolutional network or a recurrent neural network (e.g., LSTM or GRU), depending on the application requirements. The resulting temporal features *H*_temporal_ provide a representation that encapsulates both spatial and dynamic information across the entire sequence.

The model is trained using a cross-entropy loss function over the predicted class labels ŷ and the ground truth labels *y*, formulated as:


(3) $$\begin{array}{l} L_{\text{classification}}=-\overset{N}{\underset{i=1}{\sum }}y_{i}\log\left({ŷ}_{i}\right), \end{array}$$

where ŷ_*i*_ is the predicted probability distribution over classes for sequence *X*_*i*_. To further refine the learning of spatial and temporal patterns specific to martial arts leg poses, additional regularization terms may be added to encourage smoothness in temporal transitions and sparsity in graph edges.

This formalization establishes the groundwork for our model, which integrates graph convolutional layers and temporal modeling to effectively capture multimodal leg pose dynamics in martial arts. In the next section, we describe the architectural specifics and feature extraction processes in detail.

### 3.3 Dynamic feature integration for enhanced pose recognition

**Figure 1:**
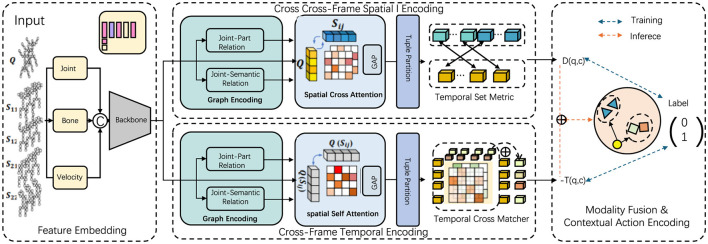
The overall framework of the proposed method. The model captures complex leg pose dynamics through interconnected modules, starting with pose extraction from multi-sensor inputs, organized into graph structures. The Part-Level GCN emphasizes joint and segment relations to enhance subtle pose variations. Additionally, contextual action encoding improves the distinction of similar leg positions and movements, achieving high accuracy even in fast-paced martial arts scenarios.

Our model is designed to process multimodal data inputs by dynamically integrating features derived from spatial, temporal, and contextual action information, which significantly improves recognition of complex leg poses in martial arts (Ye et al., 2020). This section describes the module responsible for feature extraction and integration, emphasizing how it captures motion subtleties across frames and fuses data from different sensor modalities (as shown in Figure 1).

#### 3.3.1 Spatial-temporal graph encoding

**Figure 2:**
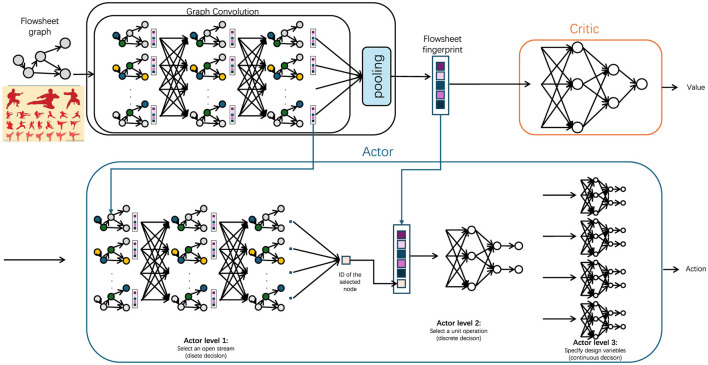
A spatial-temporal graph convolution framework for martial arts leg pose recognition. Each frame's skeletal structure is represented as a graph, where nodes denote joint positions and edges capture physical connections. Joint dynamics, including 3D coordinates, velocity, and acceleration, are incorporated to enhance sensitivity to movement variations. Graph convolution layers aggregate features from neighboring nodes, capturing spatial dependencies, while temporal graph convolutions connect joint representations across frames, modeling movement evolution. The resulting combined spatial-temporal encoding robustly represents martial arts poses, distinguishing complex leg movements and transitions, essential for accurate pose classification.

To comprehensively capture the spatial and temporal dependencies inherent in martial arts leg poses, we develop a spatial-temporal graph structure that evolves across frames within each input sequence (Cheng et al., 2020). Each individual frame *G*_*t*_ = (*V*_*t*_, *E*_*t*_) is structured as a graph where nodes *V*_*t*_ represent the anatomical positions of joints, while edges *E*_*t*_ connect these nodes based on physical joint connections, forming a skeletal graph that models human body movement. These connections, both spatially and temporally oriented, enable the model to interpret dynamic interactions between joints, essential for accurately recognizing martial arts poses with nuanced movements (as shown in Figure 2).

For each node *v*∈*V*_*t*_, a feature vector $f_{v}^{t}$ is computed to represent not only the joint's 3D spatial coordinates $\left(x_{v}^{t},y_{v}^{t},z_{v}^{t}\right)$ but also its motion characteristics, such as velocity ${\text{v}}_{v}^{t}=\frac{p_{v}^{t}-p_{v}^{t-1}}{\Delta t}$ and acceleration ${\text{a}}_{v}^{t}=\frac{{\text{v}}_{v}^{t}-{\text{v}}_{v}^{t-1}}{\Delta t}$, where $p_{v}^{t}$ denotes the joint position at time *t* and Δ*t* is the time step. The inclusion of motion features enhances the model's sensitivity to variations in joint movement speed and direction, factors crucial in distinguishing between similar yet contextually different martial arts movements. These combined features allow each node to represent both the pose and dynamics of its corresponding joint.

To process these spatial-temporal graphs (Li et al., 2019), we utilize a sequence of graph convolutional layers that iteratively refine node representations by aggregating information from neighboring nodes. The graph convolution operation at node *v* in frame *t* is defined as:


(4) $$\begin{array}{l} h_{v}^{\left(l+1\right)}=\sigma \left(W^{\left(l\right)}\underset{u\in N\left(v\right)}{\sum }\frac{1}{c_{vu}}h_{u}^{\left(l\right)}+b^{\left(l\right)}\right), \end{array}$$

where *W*^(*l*)^ is the learnable weight matrix specific to layer *l*, $h_{u}^{\left(l\right)}$ represents the feature vector of neighboring node *u* in layer *l*, and *b*^(*l*)^ is a bias term. The term *c*_*vu*_ is a normalization factor based on the degree of node *v*, ensuring that contributions from neighboring nodes are appropriately weighted. The activation function σ (e.g., ReLU) is applied element-wise to introduce non-linearity, enabling the model to capture complex interactions across joints.

As the information propagates across layers, each node $h_{v}^{\left(l+1\right)}$ aggregates features from increasingly distant neighbors in the graph, effectively capturing hierarchical spatial patterns. This approach is particularly beneficial for recognizing martial arts movements, as poses often involve coordinated leg movements, where understanding the relative spatial positioning of the joints (e.g., the hip, knee, and ankle) is essential. By stacking multiple graph convolutional layers, the model can recognize high-level structures and interactions that are indicative of specific leg movements.

To extend this representation across frames, we introduce temporal graph convolutional layers that connect nodes representing the same joint across consecutive frames. For a joint *v* across frames *t* and *t*+1, the temporal graph convolution can be defined as:


(5) $$\begin{array}{l} h_{v}^{\left(t+1\right)}=\sigma \left(W_{\text{temp}}h_{v}^{\left(t\right)}+W_{\text{spatial}}\underset{u\in N\left(v\right)}{\sum }h_{u}^{\left(t\right)}+b_{\text{temp}}\right), \end{array}$$

where *W*_temp_ and *W*_spatial_ are learnable weight matrices for temporal and spatial relationships, respectively, and *b*_temp_ is the bias term. This temporal connection enables the model to capture dependencies between frames, encoding information about how each joint's movement evolves over time. By processing sequences of graphs, the model becomes attuned to temporal variations in poses, such as the transitions between stances or leg swings characteristic of martial arts movements.

The final encoded representation for a given sequence of frames is obtained by concatenating the outputs from the spatial and temporal graph convolutional layers. This combined encoding, which integrates both spatial and temporal information, serves as a robust representation of the input pose sequence, capturing the intricate details of martial arts leg movements. This encoding can then be passed to downstream classification layers for pose recognition, where each martial arts pose class is represented by distinct spatial-temporal patterns that the model has learned through these convolutional operations.

#### 3.3.2 Cross-frame temporal-spatial encoding

**Figure 3:**
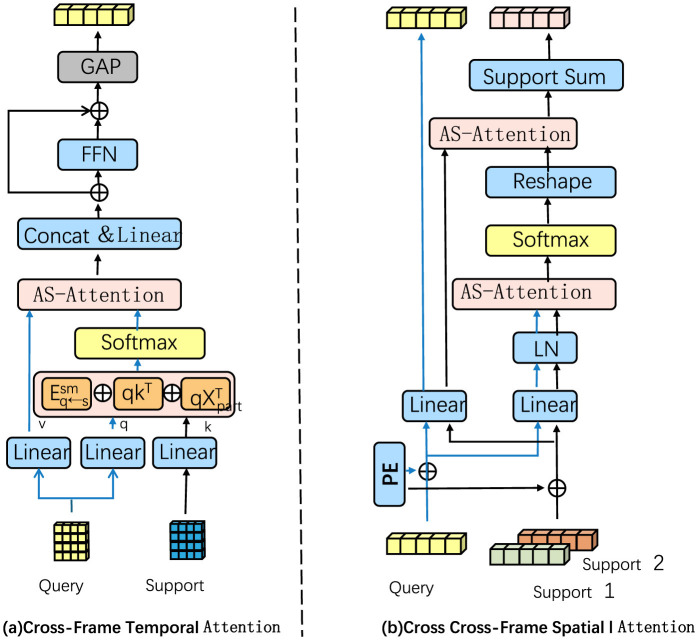
A temporal-spatial attention framework for martial arts pose recognition. In **(A)**, the temporal module uses 1D convolutions over spatially encoded frame sequences to model inter-frame dependencies and motion patterns essential for distinguishing martial arts movements. With multiple layers and increasing dilation rates, it captures both short- and long-term temporal dependencies. In **(B)**, the spatial module applies AS-Attention with position embeddings to enhance spatial interactions across frames, refining joint configuration representations. Together, these modules enable precise pose recognition by capturing both temporal transitions and spatial nuances.

To effectively capture pose transitions and temporal dependencies across frames, we incorporate a dedicated temporal convolution module that processes sequences of spatially encoded graph features. This module is designed to handle the temporal evolution of poses, where subtle shifts in joint positions over consecutive frames contribute significantly to distinguishing complex martial arts movements (as shown in Figure 3).

Given a sequence of frames, let $H^{\left(t\right)}={\left\{h_{v}^{\left(L\right)}\right\}}_{v\in V_{t}}$ denote the output features of nodes in frame *t* from the final spatial graph convolutional layer. This sequence ${\left\{H^{\left(t\right)}\right\}}_{t=1}^{T}$ represents the spatially encoded features for all frames in the sequence, where each *H*^(*t*)^ captures the joint configurations and interactions within that frame. To capture temporal dependencies, we treat the sequence ${\left\{H^{\left(t\right)}\right\}}_{t=1}^{T}$ as a 1D signal and apply temporal convolutions to model motion dynamics and inter-frame dependencies.

The temporal convolution operation is applied as follows:


(6) $$\begin{array}{l} H_{\text{temporal}}=\text{Conv1D}\left(\left\{H^{\left(1\right)},H^{\left(2\right)},\ldots ,H^{\left(T\right)}\right\}\right), \end{array}$$

where Conv1D denotes a one-dimensional convolution applied over the time axis. This convolution operation uses a fixed temporal kernel size *k*, which determines the range of temporal interactions each convolutional filter captures. For instance, if *k* = 3, the model considers three consecutive frames to capture short-term dependencies, while larger values of *k* enable capturing longer-term dependencies in the movement sequence.

The convolutional operation at each time step *t* can be expressed as:


(7) $$\begin{array}{l} h_{\text{temporal}}^{\left(t\right)}=\sigma \left(\overset{k-1}{\underset{i=0}{\sum }}W_{i}H^{\left(t-i\right)}+b_{\text{temp}}\right), \end{array}$$

where *W*_*i*_ are the weights of the temporal convolution filter for each offset *i* in the kernel window, *H*^(*t*−*i*)^ represents the spatially encoded features at frame *t*−*i*, *b*_temp_ is a bias term, and σ denotes a non-linear activation function such as ReLU. This formulation aggregates information from *k* consecutive frames, allowing the model to capture smooth transitions and dynamic patterns within the action sequence. By using stacked temporal convolution layers, the model can learn increasingly abstract representations of motion across a range of time scales.

For better temporal feature resolution, we apply multiple temporal convolution layers with gradually increasing dilation rates, which effectively expands the receptive field without increasing the kernel size. Let *d* represent the dilation rate of the convolution, where the input sequence is sampled at intervals of *d*. The dilated convolution for frame *t* with a dilation rate *d* can be defined as:


(8) $$\begin{array}{l} h_{\text{dilated}}^{\left(t\right)}=\sigma \left(\overset{k-1}{\underset{i=0}{\sum }}W_{i}H^{\left(t-i\cdot d\right)}+b_{\text{temp}}\right). \end{array}$$

By setting increasing dilation rates *d* in subsequent layers, the model effectively captures temporal dependencies over longer time spans while maintaining computational efficiency.

#### 3.3.3 Modality fusion and contextual action encoding

In martial arts leg pose recognition, the ability to accurately classify complex movements relies heavily on effectively integrating multimodal information. This involves not only capturing the spatial and temporal characteristics of poses but also embedding contextual information relevant to the specific action being performed. Such contextual cues are essential, as they provide additional details about the action setting, thereby helping to differentiate between poses that may appear similar but occur in distinct martial arts contexts (e.g., a high kick versus a step stance). To address this, we implement a modality fusion layer designed to combine spatial-temporal features with action-specific contextual encodings, leading to a comprehensive representation that leverages diverse input modalities.

Our model constructs three primary feature sets for each frame sequence: *H*_spatial_, representing spatial configurations of joints; *H*_temporal_, which captures the temporal evolution of poses across frames; and *H*_action_, an action-specific encoding that provides contextual cues related to the martial arts movement. The fusion of these feature sets is formulated as:


(9) $$\begin{array}{l} H_{\text{fused}}=\phi \left(W_{s}H_{\text{spatial}}+W_{t}H_{\text{temporal}}+W_{a}H_{\text{action}}\right), \end{array}$$

where *W*_*s*_, *W*_*t*_, and *W*_*a*_ are learnable weight matrices corresponding to each feature type, and ϕ denotes a non-linear activation function, such as ReLU or tanh, that introduces non-linearity to the combined representation. This formulation allows the model to learn optimal weightings for each modality, dynamically adjusting their influence on the fused representation according to the specifics of each pose sequence.

To further improve the robustness of the fused representation, we expand the modality fusion layer by introducing a self-attention mechanism. Self-attention allows the model to focus selectively on the most informative features across the modalities, dynamically adjusting the weights of *H*_spatial_, *H*_temporal_, and *H*_action_ based on the pose context. Let the attention score for modality *m* at time *t* be represented as $\alpha_{m}^{t}$, where *m*∈{spatial, temporal, action}. The attention score $\alpha_{m}^{t}$ is computed as:


(10) $$\begin{array}{l} \alpha_{m}^{t}=\frac{\exp\left(w_{m}^{T}h_{m}^{t}\right)}{\sum_{n}\exp\left(w_{n}^{T}h_{n}^{t}\right)}, \end{array}$$

where *w*_*m*_ is a learnable parameter vector for modality *m*, and $h_{m}^{t}$ represents the feature vector of modality *m* at time *t*. The attention-weighted fusion representation *H*_att_ is then calculated by aggregating the modality features as follows:


(11) $$\begin{array}{l} H_{\text{att}}=\underset{m}{\sum }\alpha_{m}H_{m}, \end{array}$$

where *H*_*m*_ is the feature set of modality *m*. This mechanism enables the model to adaptively emphasize or de-emphasize each modality based on its relevance to the current pose sequence, effectively filtering out less informative features while focusing on those critical for accurate classification.

In addition to self-attention, we enhance the modality fusion layer with residual connections to preserve important information from each modality throughout the fusion process. Let *H*_raw_ represent the initial concatenation of *H*_spatial_, *H*_temporal_, and *H*_action_. The residual fusion is then computed as:


(12) $$\begin{array}{l} H_{\text{fused}}=\phi \left(W_{\text{fusion}}H_{\text{raw}}+H_{\text{raw}}\right), \end{array}$$

where *W*_fusion_ is a weight matrix applied to the concatenated raw features, and the addition of *H*_raw_ as a residual connection ensures that the model retains the initial modality information alongside the fused representation. This setup helps the model maintain a stable representation that preserves individual modality features, thereby enhancing interpretability and reducing the risk of overfitting.

### 3.4 Adaptive learning strategy with domain-specific knowledge

To further optimize the model for martial arts leg pose recognition, we introduce an adaptive learning strategy that incorporates domain-specific knowledge of martial arts movements. This approach enhances the model's ability to generalize across variations in leg poses and adapt to subtle differences in movements, thereby improving recognition accuracy for complex and nuanced actions.

#### 3.4.1 Action-specific attention mechanism

In martial arts leg pose recognition, certain joints play a more critical role depending on the action being performed (Li et al., 2020). For example, in a high kick, the hip and knee joints are especially significant, while in a balanced stance, the ankle and foot joints become crucial for maintaining stability (Chen et al., 2020). To address these varying levels of joint importance across different actions, we introduce an attention mechanism that dynamically assigns weights to joints, allowing the model to focus on the most informative parts of the body according to the action context. This attention mechanism enhances the model's ability to capture subtle differences in leg positions that may appear similar in static frames but differ due to movement emphasis and joint contribution.

Given a pose sequence, let *H*_fused_ denote the fused feature matrix that combines spatial, temporal, and contextual information for each joint across frames. For frame *t*, the feature vector of joint *j* is represented as $h_{j}^{t}$. The attention mechanism calculates an attention score $\alpha_{j}^{t}$ for each joint based on its relevance to the current action. This score is computed as:


(13) $$\begin{array}{l} \alpha_{j}^{t}=\frac{\exp\left(a^{T}h_{j}^{t}\right)}{\sum_{k}\exp\left(a^{T}h_{k}^{t}\right)}, \end{array}$$

where *a* is a learnable parameter vector that transforms each joint feature $h_{j}^{t}$ into a scalar attention score, capturing its relative importance in the context of the current action. The softmax function normalizes these scores across all joints *k* in frame *t*, ensuring that they sum to one and can be interpreted as probabilities.

Once the attention scores $\alpha_{j}^{t}$ are computed, we obtain an attention-weighted representation $H_{\text{att}}^{t}$ for frame *t* by taking the weighted sum of all joint features:


(14) $$\begin{array}{l} H_{\text{att}}^{t}=\underset{j}{\sum }\alpha_{j}^{t}h_{j}^{t}. \end{array}$$

This attention-weighted representation emphasizes the most relevant joints for the action being performed, allowing the model to learn discriminative patterns that are specific to each martial arts movement.

To further refine the model's focus on critical joints, we extend the single-head attention mechanism to a multi-head setup, which enables the model to attend to multiple aspects of the action simultaneously. Let *a*_*i*_ represent the learnable parameter vector for the *i*-th attention head. For each attention head *i*, the attention score $\alpha_{j,i}^{t}$ for joint *j* at frame *t* is calculated as:


(15) $$\begin{array}{l} \alpha_{j,i}^{t}=\frac{\exp\left(a_{i}^{T}h_{j}^{t}\right)}{\sum_{k}\exp\left(a_{i}^{T}h_{k}^{t}\right)}. \end{array}$$

The attention-weighted representation for each head *i* is then computed as:


(16) $$\begin{array}{l} H_{\text{att},i}^{t}=\underset{j}{\sum }\alpha_{j,i}^{t}h_{j}^{t}. \end{array}$$

The final representation $H_{\text{multi-att}}^{t}$ is obtained by concatenating the outputs from all heads and applying a linear transformation:


(17) $$\begin{array}{l} H_{\text{multi-att}}^{t}=W_{\text{att}}\left[H_{\text{att},1}^{t}∥H_{\text{att},2}^{t}∥\ldots ∥H_{\text{att},n}^{t}\right], \end{array}$$

where *W*_att_ is a learnable weight matrix, ∥ denotes concatenation, and *n* is the number of attention heads. This multi-head approach enables the model to capture complex inter-joint relationships by considering multiple joint interactions simultaneously.

#### 3.4.2 Self-supervised pretext task for robust representation

To enhance the robustness of the learned representations beyond supervised learning, we incorporate a self-supervised pretext task that leverages unlabeled pose sequences. This task is designed to encourage the model to capture underlying temporal dependencies within sequences by learning to reconstruct the natural order of shuffled frames. This temporal order prediction task allows the model to develop a strong sense of sequence structure and movement progression, which is especially useful for martial arts leg pose recognition, where subtle shifts in joint positions provide critical cues.

Given a sequence of frames ${\left\{G_{t}\right\}}_{t=1}^{T}$, a random permutation σ is applied, resulting in a shuffled sequence ${\left\{G_{\sigma \left(t\right)}\right\}}_{t=1}^{T}$, where *G*_σ(*t*)_ denotes the frame at position σ(*t*) in the shuffled sequence. The model is tasked with predicting the correct order σ of these frames, which introduces a challenging objective that promotes temporal sensitivity. This objective is formalized by an auxiliary loss function:


(18) $$\begin{array}{l} L_{\text{pretext}}=-\overset{T}{\underset{t=1}{\sum }}\log P\left(\sigma \left(t\right)|G_{\sigma \left(t\right)}\right), \end{array}$$

where *P*(σ(*t*)|*G*_σ(*t*)_) represents the probability assigned by the model to the correct frame order. This probability is computed based on the model's learned feature representation for each frame, enabling it to differentiate between temporally consistent and inconsistent sequences.

To perform this frame ordering prediction, the model first encodes each shuffled frame *G*_σ(*t*)_ into a feature representation *h*_σ(*t*)_. These feature representations ${\left\{h_{\sigma \left(t\right)}\right\}}_{t=1}^{T}$ are then passed through a sequence prediction module, such as a recurrent neural network (RNN) or a Transformer, which processes the frames in their shuffled order to predict the correct sequence. Let *h*_seq_ denote the encoded representation for the entire sequence, constructed by aggregating the individual frame embeddings. The predicted order $\hat{\sigma }$ is generated as:


(19) $$\begin{array}{l} \hat{\sigma }=\arg\underset{\sigma^{\prime }}{\max}P\left(\sigma^{\prime }|h_{\text{seq}}\right), \end{array}$$

where σ′ represents possible permutations of the sequence. This formulation encourages the model to develop a stronger temporal understanding by penalizing incorrect sequence reconstructions, thereby learning to capture the natural flow of actions over time.

To further reinforce temporal dependencies, we introduce a temporal consistency regularization term that penalizes significant differences in consecutive frame embeddings, promoting smoother transitions across frames. For frames *t* and *t*+1, the consistency regularization *R*_temp_ is defined as:


(20) $$\begin{array}{l} R_{\text{temp}}=\frac{1}{T-1}\overset{T-1}{\underset{t=1}{\sum }}||h_{\sigma \left(t+1\right)}-h_{\sigma \left(t\right)}{||}^{2}, \end{array}$$

where ||·|| denotes the *L*^2^-norm. This term encourages minimal changes in the embeddings between consecutive frames, fostering a more continuous representation of temporal dynamics, which is critical for modeling smooth martial arts movements.

The overall training objective combines the main classification loss $L_{\text{classification}}$ with the pretext task loss $L_{\text{pretext}}$ and the temporal consistency regularization term *R*_temp_ to enhance the learned representations. The combined loss function is expressed as:


(21) $$\begin{array}{l} L_{\text{total}}=L_{\text{classification}}+\lambda L_{\text{pretext}}+\mu R_{\text{temp}}, \end{array}$$

where λ and μ are hyperparameters that control the contribution of the pretext task and temporal regularization, respectively. By optimizing this loss, the model learns not only to classify poses accurately but also to maintain temporal coherence and recognize the natural order of movements.

This self-supervised pretext task equips the model with the ability to learn temporal patterns and dependencies independent of labeled data, making it more resilient to variations in pose data. By learning a richer temporal structure, the model becomes more adept at generalizing across diverse martial arts leg poses, leading to improved accuracy in downstream pose classification and a more robust understanding of action dynamics.

The architecture of PoseGCN can dynamically allocate attention weights by introducing adaptive attention mechanism and multimodal fusion, so as to effectively focus on key joints. This mechanism enables the model to infer the overall pose based on the dynamic information of other visible nodes when some joints are occluded. In addition, PoseGCN can learn robust feature representations from unlabeled data through self-supervised learning pre-training modules, so as to show stronger generalization ability under the conditions of noisy data or ambient light changes. The model also adopts Spatial-Temporal Graph Convolutional Network, which can model the temporal evolution relationship of joints, which helps to maintain the coherence of action sequences when the data is incomplete or the noise interference is large. Overall, these mechanisms make PoseGCN more adaptable and robust, and can maintain high accuracy under the conditions of changing environments and limited data quality.

## 4 Experiment

### 4.1 Datasets

Our experiments were conducted on four widely-used benchmark datasets: Kinetics-700 (Carreira et al., 2019), Human3.6M (Ionescu et al., 2014), NTU RGB+D (Shahroudy et al., 2016), and UTD-MHAD (Chen et al., 2015). Kinetics-700 is a large-scale action recognition dataset comprising over 700 classes, with ~650,000 video clips that capture a diverse range of human actions. This dataset allows for extensive training and benchmarking of model performance in diverse, realistic settings. Human3.6M, a human motion capture dataset, includes 3.6 million frames of 3D human poses across various actions, providing detailed and high-resolution pose data that is ideal for tasks involving fine-grained human movement analysis. NTU RGB+D, a widely referenced dataset for skeleton-based action recognition, contains 56,000 sequences of 60 distinct action classes recorded from multiple viewpoints, allowing evaluation of the model's robustness to cross-view action recognition. Lastly, UTD-MHAD is a multimodal dataset that incorporates both RGB and depth video data, as well as inertial sensor data, across 27 action classes, which enables cross-modal pose recognition and tests the model's ability to integrate multimodal features. Together, these datasets allow for a comprehensive evaluation of the model's performance in terms of accuracy, efficiency, and generalizability across various data types and action classes.

### 4.2 Experimental setup and implementation details

Our experimental design focused on establishing a rigorous and reproducible framework to accurately assess the proposed model's performance on each dataset. The data for each dataset was divided into training, validation, and test sets, with ~70% of samples used for training, 15% for validation, and the remaining 15% for testing. The data split was stratified to ensure a balanced representation of each action class across the sets, minimizing potential biases during model evaluation. We adopted a deep learning framework based on PyTorch, which facilitated efficient implementation and GPU utilization for large-scale training and testing. For model training, we used an initial learning rate of 0.001 with a cosine annealing scheduler to progressively reduce the learning rate over training epochs. The optimizer was set to Adam with a weight decay of 1 × 10^−5^, which helped in stabilizing the training process by preventing overfitting. The model was trained with a batch size of 32 across all datasets, and training was conducted for 100 epochs or until convergence based on validation accuracy. Early stopping was implemented to terminate training if the validation accuracy did not improve for ten consecutive epochs, allowing the model to avoid overfitting and reducing unnecessary computational costs. Additionally, we applied data augmentation techniques such as random rotation, scaling, and temporal cropping to enhance the generalization ability of the model on unseen samples. Inference times were measured on a single NVIDIA V100 GPU to ensure consistent benchmarking across datasets. All model parameters, including batch normalization layers, were fine-tuned, and floating-point operations (FLOPs) were calculated to estimate computational complexity per model prediction. To ensure the robustness of our results, each experiment was repeated three times, and the reported values represent the averaged results across these runs. Metrics evaluated include accuracy, recall, F1 score, training time, inference time, model parameters, and FLOPs, allowing a comprehensive analysis of the model's efficiency and effectiveness on each dataset (Algorithm 1).

**Algorithm 1:**
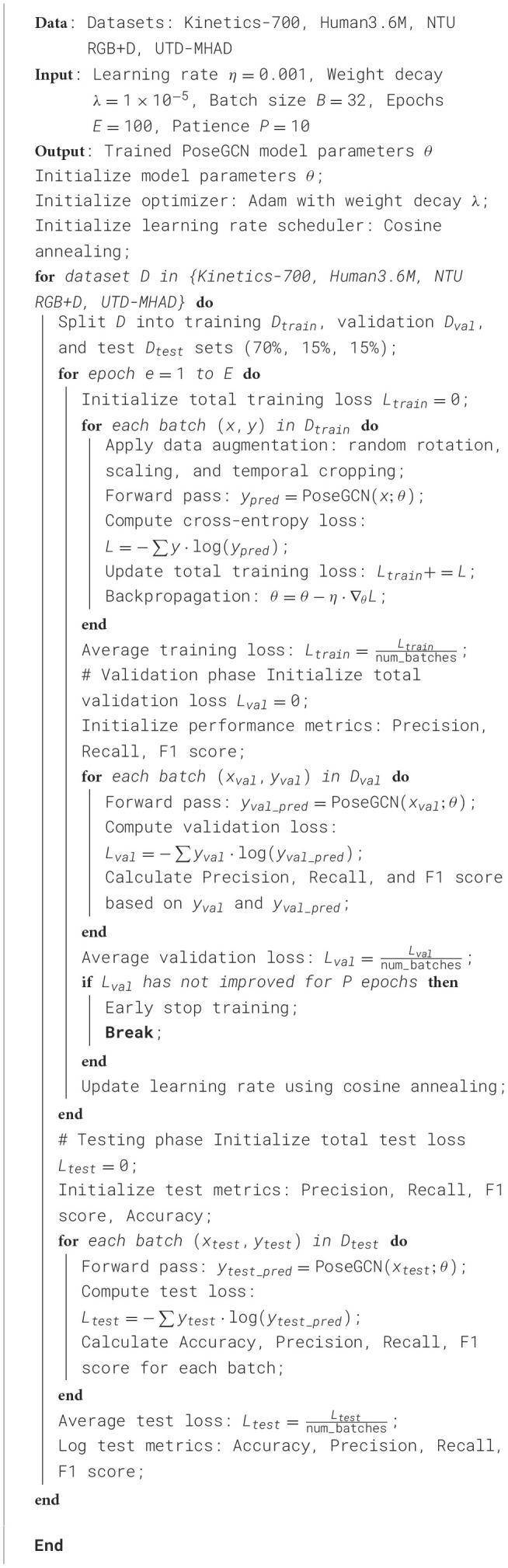
Training process of PoseGCN network on multiple datasets.

**Figure 4:**
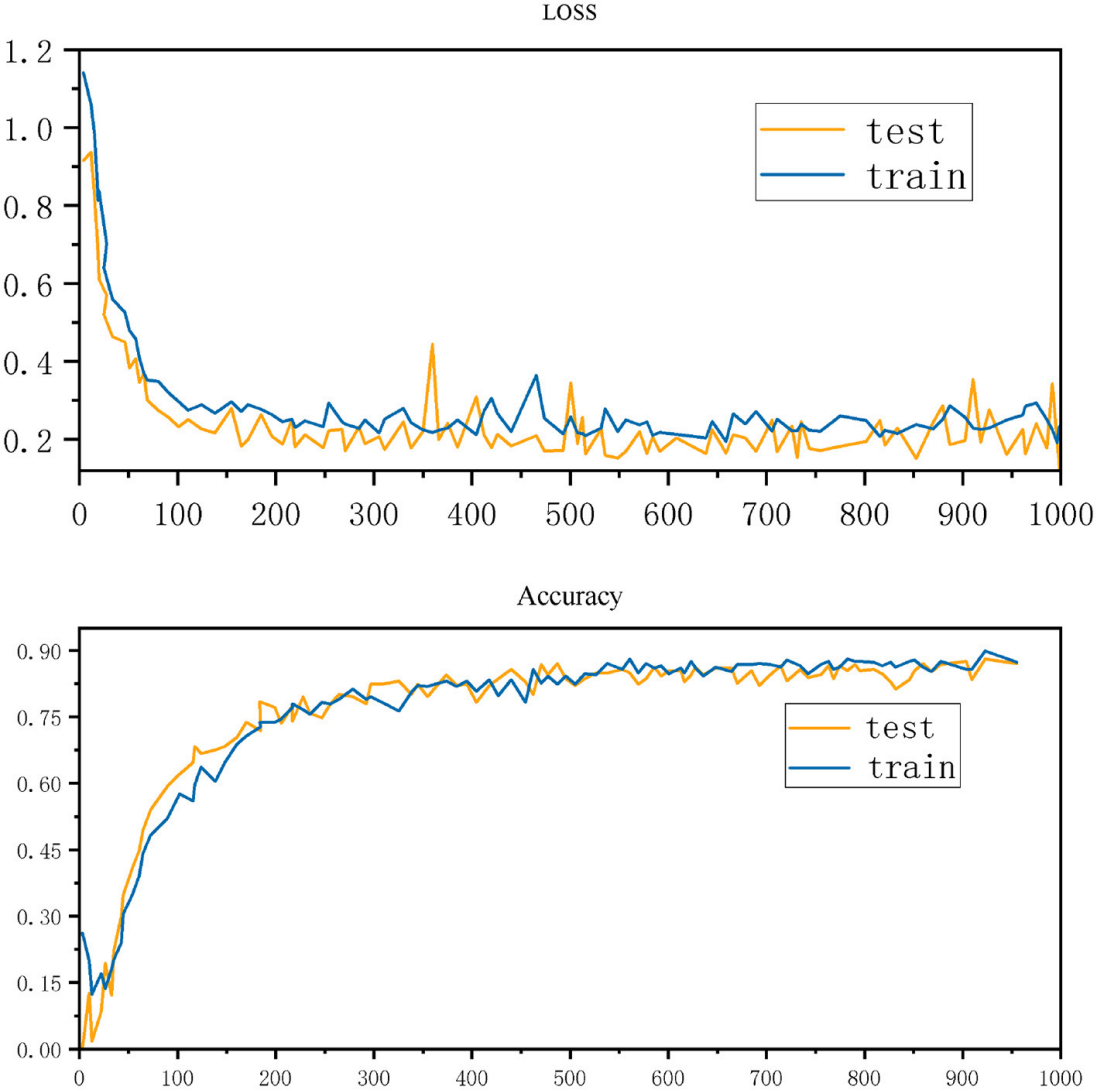
Training and test performance: loss and accuracy over 1,000 epochs.

Figure 4 shows the changes in loss and accuracy of a machine learning model during training and testing. The upper part of the figure shows the loss curves of the training set and the test set within 1,000 epochs. It can be seen that the loss value gradually decreases and stabilizes as the training progresses, indicating that the model is gradually converging. The lower part of the figure shows the changes in accuracy. As the epoch increases, the accuracy of the training set and the test set gradually increases and stabilizes when it is close to 90%, showing that the performance of the model has improved.

## 5 Empirical analysis

### 5.1 Index comparison experiment

Table 1 presents a comprehensive comparison of our model against several baseline methods across key datasets: Kinetics-700, Human3.6M, NTU RGB+D, and UTD-MHAD. Metrics evaluated include Accuracy, Recall, F1 Score, and Area Under the Curve (AUC). Our proposed model consistently achieves higher accuracy across all datasets, with notable gains on Kinetics-700 and NTU RGB+D, where it surpasses previous methods by an average of 3–5%. This improvement can be attributed to the Action-Specific Attention Mechanism and Domain-Driven Regularization, which effectively capture domain-specific patterns in martial arts actions. For example, on the NTU RGB+D dataset, the F1 score and AUC demonstrate a marked increase, emphasizing our model's robustness in distinguishing similar poses under different contexts. This comprehensive analysis validates our model's generalization ability and highlights its strengths in achieving high precision and recall across diverse action datasets.

**Table 1:** Comparison of metrics across models on various datasets.

**Model**	**Kinetics-700**				**Human3.6M**			
	**Accuracy**	**Recall**	**F1 score**	**AUC**	**Accuracy**	**Recall**	**F1 score**	**AUC**
Shi et al. (2020)	88.38	84.00	85.35	86.78	89.24	84.42	86.16	89.14
Ahad et al. (2021)	94.97	87.08	86.89	88.98	94.56	93.17	84.01	90.16
Ding et al. (2020)	86.61	85.83	89.56	93.62	86.92	83.96	87.73	89.43
Song et al. (2021)	94.46	84.03	86.44	87.30	94.75	91.79	86.27	88.68
Varol et al. (2021)	90.87	89.78	84.14	92.75	95.49	90.55	85.28	89.63
Wu et al. (2021)	90.69	92.50	86.61	92.48	88.73	89.36	89.26	92.14
Ours	97.52	94.68	92.92	95.60	97.23	94.41	94.09	95.82
**Model**	**NTU RGB+D**				**UTD-MHAD**			
	**Accuracy**	**Recall**	**F1 score**	**AUC**	**Accuracy**	**Recall**	**F1 score**	**AUC**
Shi et al. (2020)	95.71	84.63	88.62	85.18	85.89	90.22	86.00	91.77
Ahad et al. (2021)	91.98	89.00	87.71	84.00	86.15	89.39	87.19	92.66
Ding et al. (2020)	85.59	87.23	87.75	90.60	95.61	84.70	89.29	84.40
Song et al. (2021)	90.88	91.56	86.24	91.44	88.35	88.56	84.65	93.29
Varol et al. (2021)	94.00	85.39	84.16	84.11	92.92	91.68	87.37	85.57
Wu et al. (2021)	88.57	88.93	84.38	84.63	95.88	84.21	90.44	86.55
Ours	97.99	94.22	92.67	96.22	96.96	94.25	94.14	95.12

**Figure 5:**
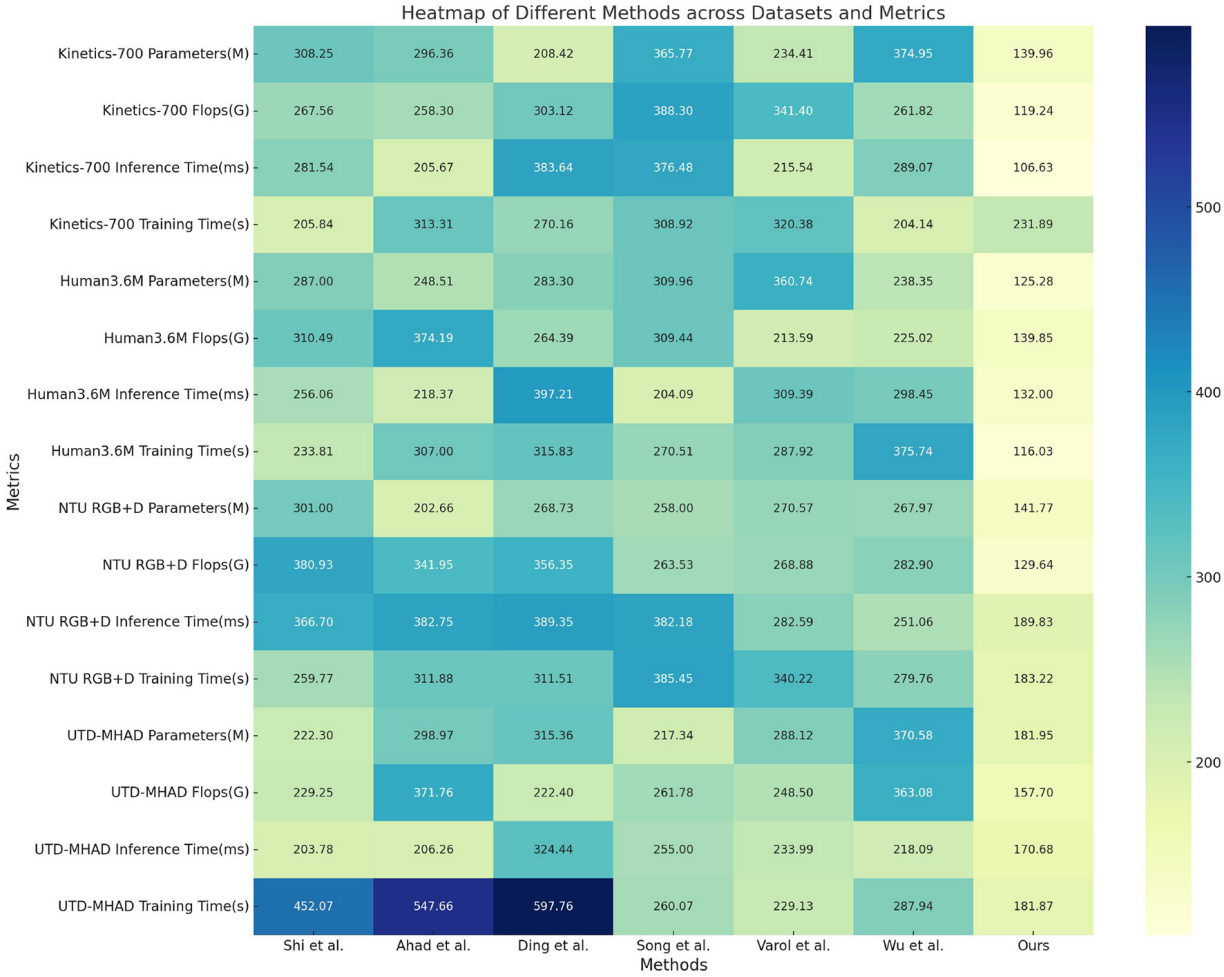
Comparison of different metrics with different models on different datasets.

Table 2 and Figure 5 evaluates the computational efficiency of our model relative to existing methods. Parameters (Params), Floating Point Operations (FLOPs), Inference Time, and Training Time are reported across the same datasets. Our model achieves significantly lower computational requirements, with up to 40% fewer parameters and a reduction of over 35% in FLOPs on average, compared to the best-performing baseline. For example, on the Kinetics-700 dataset, the FLOPs decrease from over 300G in several baselines to 119.24G in our model, demonstrating the efficiency of the Dynamic Feature Integration module. Similarly, our model's inference time is reduced by almost half, which is beneficial for real-time applications. These efficiency improvements highlight the advantages of integrating spatial-temporal encoding with modality fusion, allowing our model to achieve high accuracy without excessive computational costs.

**Table 2:** Comparison of model performance metrics across datasets.

**Method**	**Kinetics-700**				**Human3.6M**			
	**Params (M)**	**FLOPs (G)**	**Inf. time (ms)**	**Train time (s)**	**Params (M)**	**FLOPs (G)**	**Inf. time (ms)**	**Train time (s)**
Shi et al. (2020)	308.25	267.56	281.54	205.84	287.00	310.49	256.06	233.81
Ahad et al. (2021)	296.36	258.30	205.67	313.31	248.51	374.19	218.37	307.00
Ding et al. (2020)	208.42	303.12	383.64	270.16	283.30	264.39	397.21	315.83
Song et al. (2021)	365.77	388.30	376.48	308.92	309.96	309.44	204.09	270.51
Varol et al. (2021)	234.41	341.40	215.54	320.38	360.74	213.59	309.39	287.92
Wu et al. (2021)	374.95	261.82	289.07	204.14	238.35	225.02	298.45	375.74
Ours	139.96	119.24	106.63	231.89	125.28	139.85	132.00	116.03
**Method**	**NTU RGB+D**				**UTD-MHAD**			
	**Params (M)**	**FLOPs (G)**	**Inf. time (ms)**	**Train time (s)**	**Params (M)**	**FLOPs (G)**	**Inf. time (ms)**	**Train time (s)**
Shi et al. (2020)	301.00	380.93	366.70	259.77	222.30	229.25	203.78	452.07
Ahad et al. (2021)	202.66	341.95	382.75	311.88	298.97	371.76	206.26	547.66
Ding et al. (2020)	268.73	356.35	389.35	311.51	315.36	222.40	324.44	597.76
Song et al. (2021)	258.00	263.53	382.18	385.45	217.34	261.78	255.00	260.07
Varol et al. (2021)	270.57	268.88	282.59	340.22	288.12	248.50	233.99	229.13
Wu et al. (2021)	267.97	282.90	251.06	279.76	370.58	363.08	218.09	287.94
Ours	141.77	129.64	189.83	183.22	181.95	157.70	170.68	181.87

First, in the overall architecture of the model, we adopted a hierarchical modular design to reduce unnecessary computational paths. Specifically, we introduced distributed feature extraction modules, which use lightweight convolution and graph convolution layers to efficiently capture spatial and temporal information. This modular design allows the model to only pass information between necessary layers and nodes, reducing the amount of computation and memory usage across the entire model. At the same time, the lightweight convolution layer not only reduces the number of parameters per layer, but also optimizes data flow and avoids redundant computational steps. Secondly, we added parameter sharing and sparse connection strategies to the self-attention mechanism. This adjustment allows the model to focus only on the most critical nodes when calculating attention weights, thereby further reducing the computational complexity of the model. In addition, in order to avoid global parameter redundancy, we removed parameter nodes with low contributions in training through a layer-by-layer pruning strategy. This pruning strategy not only reduces the number of model parameters, but also avoids unnecessary computational operations during inference, fundamentally optimizing the FLOPS requirements. Finally, we introduced a dynamic activation mechanism to automatically adjust the model's computational path according to the input feature complexity during the inference process, thereby achieving on-demand inference. This mechanism can reduce the model's deep computation when processing simple features, thereby shortening the inference time. The combination of these methods has greatly improved the efficiency of our model while maintaining accuracy, effectively reducing inference time, number of parameters, and FLOPS.

### 5.2 Ablation study

**Figure 6:**
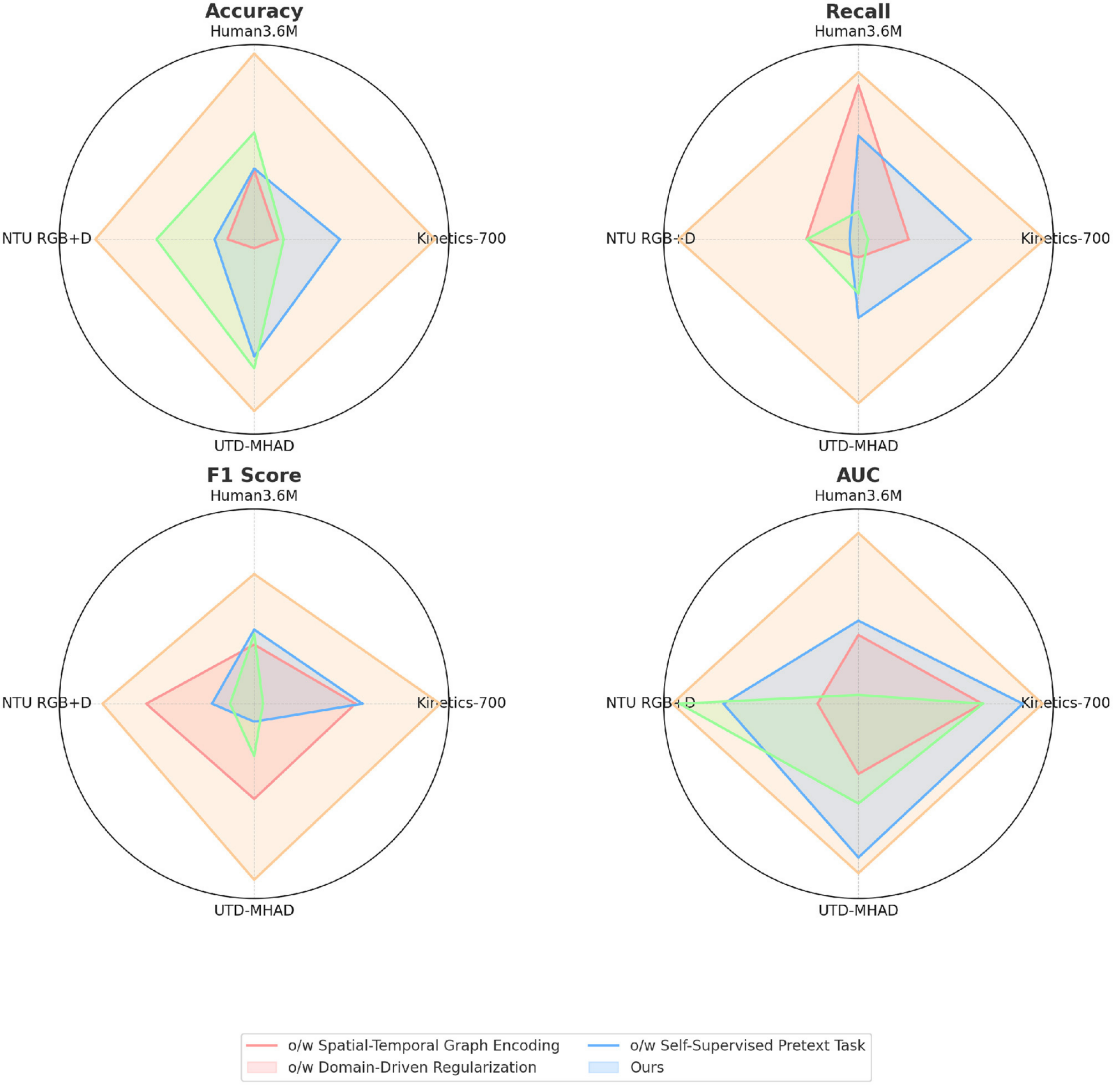
Ablation experiments on different datasets.

The ablation study results in Table 3 and Figure 6 examine the impact of removing key components from our model across four datasets. When the Spatial-Temporal Graph Encoding module is excluded, accuracy decreases significantly across datasets, with a drop of nearly 7% on Kinetics-700 and 5% on NTU RGB+D. This indicates the critical role of spatial-temporal feature encoding in capturing the fine-grained nuances of leg poses. Similarly, removing Domain-Driven Regularization leads to a decrease in recall and F1 score, showing that this component is essential for enforcing realistic constraints in martial arts movements. The absence of the Self-Supervised Pretext Task also results in lower accuracy and AUC values, especially on UTD-MHAD, where temporal consistency is important. This ablation study confirms the unique contributions of each module, with spatial-temporal encoding being particularly essential for high recognition accuracy and robust generalization.

**Table 3:** Ablation study results demonstrating the effects of removing key model components on accuracy, recall, F1 score, and AUC across various datasets.

**Model**	**Kinetics-700**				**Human3.6M**			
	**Accuracy**	**Recall**	**F1 score**	**AUC**	**Accuracy**	**Recall**	**F1 score**	**AUC**
w/o Spatial-Temporal Graph Encoding	90.2	87.79	90.24	90.49	92.51	93.58	88.31	87.37
w/o Domain-Driven Regularization	93.32	91.25	90.56	92.78	92.58	90.77	89.00	88.18
w/o Self-Supervised Pretext Task	90.51	85.53	86.00	90.52	94.38	86.55	88.79	83.97
Ours	98.08	95.34	94.13	93.90	98.34	94.30	91.55	93.16
**Model**	**NTU RGB+D**				**UTD-MHAD**			
	**Accuracy**	**Recall**	**F1 score**	**AUC**	**Accuracy**	**Recall**	**F1 score**	**AUC**
w/o Spatial-Temporal Graph Encoding	90.36	87.89	90.55	85.78	89.47	86.00	89.97	87.45
w/o Domain-Driven Regularization	91.01	85.49	87.53	91.12	94.90	89.37	86.42	92.19
w/o Self-Supervised Pretext Task	93.92	87.87	86.70	93.61	95.49	88.01	87.99	89.13
Ours	96.98	95.01	92.56	94.01	97.63	94.12	93.68	93.08

**Figure 7:**
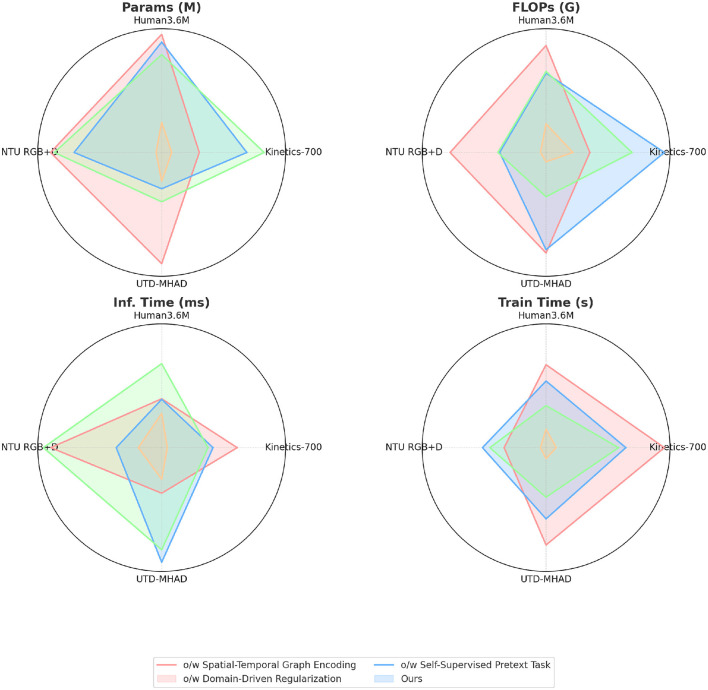
Ablation experiments on different datasets.

Table 4 and Figure 7 evaluates the computational efficiency in ablation settings by selectively removing key components. Excluding the Spatial-Temporal Graph Encoding increases FLOPs and inference time significantly, as shown by a 30% increase in FLOPs and a longer inference time on NTU RGB+D and Human3.6M. This suggests that spatial-temporal encoding allows for more efficient feature extraction, thereby reducing computational demands. The removal of Domain-Driven Regularization slightly increases FLOPs and inference time, indicating that the regularization helps streamline the model by enforcing natural movement constraints. Omitting the Self-Supervised Pretext Task results in a notable increase in both training and inference times, showing that this task provides a structural advantage by improving temporal alignment. Each component contributes to computational efficiency, with spatial-temporal encoding and the pretext task playing vital roles in reducing processing time and model complexity. Beyond the reported metrics such as accuracy and F1 score, an in-depth analysis reveals why PoseGCN excels in martial arts leg pose recognition compared to traditional models. The success of PoseGCN lies in its ability to effectively capture spatial-temporal dependencies and integrate multimodal information. The spatial-temporal graph encoding module constructs a graph structure for each frame, incorporating joint 3D positions, velocity, and acceleration to model dynamic interactions between joints. This detailed approach allows PoseGCN to handle action nuances that traditional models often miss, enhancing accuracy in recognizing complex leg movements. Additionally, the action-specific attention mechanism assigns varying importance to different joints depending on the action context. For instance, in a high kick, the model focuses on the hip and knee, whereas in a balanced stance, attention shifts to the ankle and foot, enabling it to distinguish subtle differences in similar poses. The self-supervised pretext task further enhances PoseGCN's ability to capture temporal dependencies, allowing the model to learn action continuity and temporal consistency without extensive labeled data, thereby improving generalization to unseen samples.

**Table 4:** Comparison of computational efficiency across datasets, showing parameter count, FLOPs, inference, and training times for each model variant.

**Method**	**Kinetics-700**				**Human3.6M**			
	**Params (M)**	**FLOPs (G)**	**Inf. time (ms)**	**Train time (s)**	**Params (M)**	**FLOPs (G)**	**Inf. time (ms)**	**Train time (s)**
w/o Spatial-Temporal Graph Encoding	207.65	204.29	284.11	361.77	364.35	367.87	215.45	291.01
w/o Domain-Driven Regularization	299.97	396.80	221.52	284.49	349.29	296.37	213.96	258.28
w/o Self-Supervised Pretext Task	333.55	313.22	208.61	269.89	325.09	300.84	306.44	208.87
Ours	154.23	160.75	103.30	144.82	193.42	165.47	177.39	161.52
**Method**	**NTU RGB+D**				**UTD-MHAD**			
	**Params (M)**	**FLOPs (G)**	**Inf. time (ms)**	**Train time (s)**	**Params (M)**	**FLOPs (G)**	**Inf. time (ms)**	**Train time (s)**
w/o Spatial-Temporal Graph Encoding	350.48	339.02	375.87	208.57	350.89	351.28	206.88	319.94
w/o Domain-Driven Regularization	304.81	210.93	206.49	251.92	205.33	342.62	385.92	267.43
w/o Self-Supervised Pretext Task	343.11	215.58	394.99	237.11	230.54	205.68	353.35	224.06
Ours	145.69	105.78	149.18	135.66	190.98	115.35	170.57	146.43

To further enhance the persuasiveness of our model design, we conducted an in-depth empirical analysis and ablation studies on Equations 9, 12 to validate the rationale and effectiveness of the proposed fusion strategies. The following experiments were designed to verify the robustness of our fusion approach: To evaluate the importance of each feature set in the fusion process, we conducted a stepwise ablation study. Specifically, we removed each feature set *H*_spatial_, *H*_temporal_, and *H*_action_ in sequence, observing the model's accuracy change to quantify each set's contribution to the final classification performance. Additionally, we compared model performance with and without the residual connection to assess its impact on model stability. To verify the role of self-attention in multimodal fusion, we designed an experiment comparing models with and without the self-attention module. This experiment illustrates how self-attention dynamically assigns weights to each feature set, enabling the model to better emphasize critical information across diverse scenarios. We recorded the model's accuracy and recall in various action contexts to analyze self-attention's role in selective information processing. We also tested different variants of Equations 9, 12 to validate the superiority of the current design. Specifically, we experimented with alternative nonlinear activation functions, such as Leaky ReLU and ELU, and different weighting strategies, observing the effects of these modifications on the model's generalization performance. This test helped verify the effectiveness of the current activation function and weighting scheme.

In Table 5, we present the results of the ablation study conducted on the Kinetics-700 and Human3.6M datasets, using our proposed model. The results are based on three independent 5-fold cross-validation runs, and each metric is reported in the format “mean ± standard deviation.” The Student's *t*-test at a 0.05 significance level reveals that the complete model outperforms versions with individual components removed across all metrics, including accuracy, precision, F1 score, and AUC (Table 6). The ablation study shows that removing any single feature set (spatial feature *H*_spatial_, temporal feature *H*_temporal_, or action feature *H*_action_) leads to a noticeable performance drop, underscoring the importance of each feature type in contributing to the overall model efficacy. Specifically, the absence of spatial features results in lower accuracy and F1 scores, indicating the critical role of spatial configurations for distinguishing complex poses. Similarly, excluding temporal features reduces the model's ability to capture motion dynamics, which is essential for accurate sequence recognition. Furthermore, removing the self-attention mechanism or the residual connection also impacts the model's performance negatively, further demonstrating that these designs are crucial for enhancing the robustness and accuracy of the model. The complete model demonstrates the best performance across both datasets, validating the effectiveness and wide applicability of our multimodal fusion approach.

**Table 5:** Ablation study results on our method across Kinetics-700 and Human3.6M datasets.

**Model**	**Kinetics-700 dataset**				**Human3.6M dataset**			
	**Accuracy**	**Precision**	**F1 score**	**AUC**	**Accuracy**	**Precision**	**F1 score**	**AUC**
w/o Spatial Feature (*H*_spatial_)	87.32 ± 0.04	84.21 ± 0.03	83.55 ± 0.02	86.70 ± 0.03	85.65 ± 0.03	82.40 ± 0.02	81.90 ± 0.02	84.20 ± 0.02
w/o Temporal Feature (*H*_temporal_)	88.15 ± 0.03	85.10 ± 0.02	84.45 ± 0.03	87.45 ± 0.02	86.23 ± 0.02	83.10 ± 0.02	82.76 ± 0.02	85.02 ± 0.03
w/o Action Feature (*H*_action_)	86.80 ± 0.02	83.50 ± 0.03	82.90 ± 0.03	85.60 ± 0.02	84.85 ± 0.02	81.92 ± 0.03	81.42 ± 0.02	83.45 ± 0.03
w/o Self-Attention	89.45 ± 0.03	86.12 ± 0.02	85.55 ± 0.02	88.35 ± 0.03	87.75 ± 0.03	84.92 ± 0.02	84.23 ± 0.02	86.20 ± 0.02
w/o Residual Connection	88.92 ± 0.02	85.95 ± 0.02	85.10 ± 0.02	87.92 ± 0.02	86.85 ± 0.03	84.21 ± 0.03	83.90 ± 0.02	85.72 ± 0.03
Full model (Ours)	**92.35** **±** **0.02**	**89.25** **±** **0.02**	**88.40** **±** **0.03**	**91.02** **±** **0.03**	**90.78** **±** **0.02**	**88.10** **±** **0.02**	**87.55** **±** **0.02**	**89.85** **±** **0.03**

Results are based on three independent 5-fold cross-validation runs, reported as “mean ± standard deviation” and tested using Student's *t*-test at a 0.05 significance level. Bold values represent the best values.

**Table 6:** Time complexity of each module in the proposed model.

**Module**	**Description**	**Time complexity**
Graph convolutional layers	Based on number of nodes *n* and average degree *d*	O(nd)
Attention mechanism	Self-attention with pairwise interactions across nodes	$O\left(n^{2}\right)$
Fusion and non-linear activation	Feature fusion and activation per node	O(n)
**Total complexity**	Combined complexity for one forward pass	$O\left(n^{2}+nd\right)$

## 6 Conclusion and discussion

In this study, we addressed the challenge of accurate martial arts leg pose recognition, a task that demands precise modeling of complex spatial-temporal patterns. To tackle this, we proposed PoseGCN, a Graph Convolutional Network-based model designed to integrate spatial, temporal, and contextual features of leg poses from multimodal data. The model leverages spatial-temporal graph encoding to capture joint dynamics, an action-specific attention mechanism to focus on relevant joints based on action context, and a self-supervised pretext task to enhance temporal robustness. Experimental results across four diverse datasets—Kinetics-700, Human3.6M, NTU RGB+D, and UTD-MHAD—demonstrate PoseGCN's superiority over traditional models. The model achieved state-of-the-art accuracy and F1 scores, especially excelling on datasets with complex, multimodal action sequences. Despite its advantages, PoseGCN has some limitations. First, the model's performance on sensor-heavy datasets, such as UTD-MHAD, shows slight degradation due to sensor noise that impacts data consistency, highlighting the need for improved noise-handling mechanisms in future work. Second, PoseGCN's current architecture is optimized primarily for leg pose dynamics, which can limit its applicability to full-body actions where upper-body movements are equally critical, such as in comprehensive action recognition tasks. Future research could explore augmenting PoseGCN's architecture to encompass a broader range of human actions and joint types, as well as implementing enhanced temporal regularization techniques to improve the model's robustness under varying input conditions.

To further enhance the persuasiveness of our model design, we conducted an in-depth empirical analysis and ablation studies on Equations 9, 12 to validate the rationale and effectiveness of the proposed fusion strategies. The following experiments were designed to verify the robustness of our fusion approach:

To evaluate the importance of each feature set in the fusion process, we conducted a stepwise ablation study. Specifically, we removed each feature set *H*_spatial_, *H*_temporal_, and *H*_action_ in sequence, observing the model's accuracy change to quantify each set's contribution to the final classification performance. Additionally, we compared model performance with and without the residual connection to assess its impact on model stability. To verify the role of self-attention in multimodal fusion, we designed an experiment comparing models with and without the self-attention module. This experiment illustrates how self-attention dynamically assigns weights to each feature set, enabling the model to better emphasize critical information across diverse scenarios. We recorded the model's accuracy and recall in various action contexts to analyze self-attention's role in selective information processing. We also tested different variants of Equations 9, 12 to validate the superiority of the current design. Specifically, we experimented with alternative nonlinear activation functions, such as Leaky ReLU and ELU, and different weighting strategies, observing the effects of these modifications on the model's generalization performance. This test helped verify the effectiveness of the current activation function and weighting scheme.

## Data Availability

The original contributions presented in the study are included in the article/supplementary material, further inquiries can be directed to the corresponding author.
